# Supplementary material for: In silico-labeled ghost cytometry
Source: eLife. 2021 Dec 21;10:e67660. doi: 10.7554/eLife.67660 (PMC8691837; doi:10.7554/eLife.67660)
Supplement: Transparent reporting form [file elife-67660-transrepform1.docx]

***eLife’s* transparent reporting form**

We encourage authors to provide detailed information *within their submission* to facilitate the interpretation and replication of experiments. Authors can upload supporting documentation to indicate the use of appropriate reporting guidelines for health-related research (see [EQUATOR Network](http://www.equator-network.org/%20)), life science research (see the [BioSharing Information Resource](https://biosharing.org/)), or the [ARRIVE guidelines](http://www.plosbiology.org/article/info:doi/10.1371/journal.pbio.1000412) for reporting work involving animal research. Where applicable, authors should refer to any relevant reporting standards documents in this form.

If you have any questions, please consult our Journal Policies and/or contact us: [editorial@elifesciences.org](mailto:editorial@elifesciences.org).

**Sample-size estimation**

- You should state whether an appropriate sample size was computed when the study was being designed
- You should state the statistical method of sample size computation and any required assumptions
- If no explicit power analysis was used, you should describe how you decided what sample (replicate) size (number) to use

Please outline where this information can be found within the submission (e.g., sections or figure legends), or explain why this information doesn’t apply to your submission:

The number of training samples for the iSGC sorter was determined by the settings on the FPGA. The number of training samples for other machine learning models were determined as a number which, upon increase, did not substantially change the AUC or the macro-averaged F1-score.

**Replicates**

- You should report how often each experiment was performed
- You should include a definition of biological versus technical replication
- The data obtained should be provided and sufficient information should be provided to indicate the number of independent biological and/or technical replicates
- If you encountered any outliers, you should describe how these were handled
- Criteria for exclusion/inclusion of data should be clearly stated
- High-throughput sequence data should be uploaded before submission, with a private link for reviewers provided (these are available from both GEO and ArrayExpress)

Please outline where this information can be found within the submission (e.g., sections or figure legends), or explain why this information doesn’t apply to your submission:

The classification and sorting of different cell types were performed multiple times with different cell lines and had consistent results. The classification of live, dead, and apoptotic cells were also peformed multiple times with iPSCs as well as cell lines and had consistent results. The classification of undifferentiated and differentiated cells were performed multiple times for each cell combination, and had consistent results. The classification of RPE and retinoblastoma cells were performed only once due to the restricted availability of the RPE cells. The differential classification of WBCs was performed multiple times with peripheral blood samples from different patients and had consistent results.

Data exclusion based on FSC and SSC (BSC) were performed for all flow cytometry and iSGC data to exclude any debris or doublets and dead cells except for the analysis of live, dead, and apoptotic cells. Dead cells were also excluded by the intensity of Calcein AM for the analysis of undifferentiated and differentiated cells. When performing SVM, further exclusion of cells that are outliers were performed to obtain cells with a definite label. Also, the iSGC waveform was checked to additionally remove doublets that were not able to be excluded by FSC and SSC (BSC).

**Statistical reporting**

- Statistical analysis methods should be described and justified
- Raw data should be presented in figures whenever informative to do so (typically when N per group is less than 10)
- For each experiment, you should identify the statistical tests used, exact values of N, definitions of center, methods of multiple test correction, and dispersion and precision measures (e.g., mean, median, SD, SEM, confidence intervals; and, for the major substantive results, a measure of effect size (e.g., Pearson's r, Cohen's d)
- Report exact p-values wherever possible alongside the summary statistics and 95% confidence intervals. These should be reported for all key questions and not only when the p-value is less than 0.05.

Please outline where this information can be found within the submission (e.g., sections or figure legends), or explain why this information doesn’t apply to your submission:

The details of the analysis methods including the type of machine learning model, amount of data used for training and testing, and validation methods are described in the Materials and Methods section.

(For large datasets, or papers with a very large number of statistical tests, you may upload a single table file with tests, Ns, etc., with reference to sections in the manuscript.)

**Group allocation**

- Indicate how samples were allocated into experimental groups (in the case of clinical studies, please specify allocation to treatment method); if randomization was used, please also state if restricted randomization was applied
- Indicate if masking was used during group allocation, data collection and/or data analysis

Please outline where this information can be found within the submission (e.g., sections or figure legends), or explain why this information doesn’t apply to your submission:

No group allocation was performed.

**Additional data files (“source data”)**

- We encourage you to upload relevant additional data files, such as numerical data that are represented as a graph in a figure, or as a summary table
- Where provided, these should be in the most useful format, and they can be uploaded as “Source data” files linked to a main figure or table
- Include model definition files including the full list of parameters used
- Include code used for data analysis (e.g., R, MatLab)
- Avoid stating that data files are “available upon request”

Please indicate the figures or tables for which source data files have been provided:

For Fig. 2 to Fig. 5, source data files will be provided on Zenodo (doi: 10.5281/zenodo.5656641).
